# Tuberculosis in badgers where the bovine tuberculosis epidemic is expanding in cattle in England

**DOI:** 10.1038/s41598-021-00473-6

**Published:** 2021-10-25

**Authors:** Benjamin Michael Connor Swift, Elsa Sandoval Barron, Rob Christley, Davide Corbetta, Llorenç Grau-Roma, Chris Jewell, Colman O’Cathail, Andy Mitchell, Jess Phoenix, Alison Prosser, Catherine Rees, Marion Sorley, Ranieri Verin, Malcolm Bennett

**Affiliations:** 1 The Royal Veterinary College, North Mymms, Hertfordshire, AL9 7TA UK; 2 School of Veterinary Medicine and Science, University of Nottingham, Nottingham, LE12 5RD UK; 3 Institute of Infection, Veterinary Ecological Sciences, University of Liverpool, Liverpool, CH64 7TE UK; 4 Department of Veterinary Medicine, University of Cambridge, Cambridge, CB3 0ES UK; 5 Institute of Animal Pathology, University of Bern, Länggassstrasse 122, 3012 Bern, Switzerland; 6 Centre for Health Informatics, Computing, and Statistics, University of Lancaster, Lancaster, LA1 4YW UK; 7 Animal and Plant Health Agency (APHA), New Haw, Addlestone, KT15 3NB Surrey UK; 8 Department of Sociology, University of Lancaster, Lancaster, LA1 4YW UK; 9 School of Bioscience, University of Nottingham, Nottingham, LE12 5RD UK; 10 Dipartimento di Biomedicina Comparata e Alimentazione, Università Degli Studi di Padova, 35020 Legnaro, Padova Italy

**Keywords:** Microbiology, Tuberculosis

## Abstract

Bovine tuberculosis (bTB) is an important animal health and economic problem for the cattle industry and a potential zoonotic threat. Wild badgers (*Meles meles*) play a role on its epidemiology in some areas of high prevalence in cattle, particularly in the UK and Republic of Ireland and increasingly in parts of mainland Europe. However, little is known about the involvement of badgers in areas on the spatial edge of the cattle epidemic, where increasing prevalence in cattle is seen. Here we report the findings of a study of found-dead (mainly road-killed) badgers in six counties on the edge of the English epidemic of bTB in cattle. The overall prevalence of *Mycobacterium tuberculosis* complex (MTC) infection detected in the study area was 51/610 (8.3%, 95% CI 6.4–11%) with the county-level prevalence ranging from 15 to 4–5%. The MTC spoligotypes of recovered from badgers and cattle varied: in the northern part of the study area spoligotype SB0129 predominated in both cattle and badgers, but elsewhere there was a much wider range of spoligotypes found in badgers than in cattle, in which infection was mostly with the regional cattle spoligotype. The low prevalence of MTC in badgers in much of the study area, and, relative to in cattle, the lower density of sampling, make firm conclusions difficult to draw. However, with the exception of Cheshire (north-west of the study area), little evidence was found to link the expansion of the bTB epidemic in cattle in England to widespread badger infection.

## Introduction

Bovine tuberculosis (bTB), primarily caused by *Mycobacterium bovis*, is an important animal health and economic problem for the British cattle industry, and an important zoonotic infection globally, particularly where bovine milk, the main source of human infection, is not treated^1^. *M. bovis*, is a member of the *M. tuberculosis* complex (MTC) group of bacteria and predominantly infects cattle, but has a wide host range that includes badgers (*Meles meles*), other wildlife, camelids, goats, humans and some companion animals. Badgers are reported to act as one of the ‘reservoirs’ of *M. bovis* bacilli, at least in some areas^2–5^. *M. tuberculosis*, the overwhelmingly most frequent cause of human TB and *M. microti*, which mainly circulates in field voles (*Microtus agrestis*)^6^, are also found in Great Britain.

The incidence of TB in cattle in England and Wales has been rising since the mid-1980s, and the area in which it is considered endemic has gradually expanded to cover most of the south west of England and south Wales, the West Midlands and parts of mid-Wales^2^. Although usually described as a single spreading epidemic, the regional distributions of different spoligotypes of *M. bovis* might suggest a series of regional epidemics^7^. The government’s strategy for achieving ‘officially TB-free (OTF)’ status for England is based on geographic areas of risk, with different surveillance and control measures applied to different areas: the Low Risk Area (LRA), the Edge Area and the High Risk Area (HRA). The Edge Area is a boundary of intermediate TB incidence in cattle, situated between the HRA and LRA and, at the time of this study, comprised eleven counties (or part-counties), where surveillance suggested that the historically low incidence of TB in cattle was increasing.

The control of TB in cattle in many parts of the world is complicated by the involvement of wildlife species, such as deer^8^ and, in England, Wales, the island of Ireland and increasingly in some other European countries, badgers (*Meles meles*)^2,3^. It is unclear whether or not badgers are technically a reservoir host in the HRA (i.e. not only a source of infection to cattle—the ‘target’ host—but able to maintain infection without reintroduction from cattle)^2,9^, and even less is known about the role of badgers in cattle infection in the Edge Area. Thus the expansion of the cattle epidemic in the Edge Area could be the result of cattle-to-cattle, badger-to-badger, cross species transmission, or, indeed, other even less well-understood transmission routes such as through soil and farm waste^10–13^; and the relative contributions of each potential transmission route, or if they differ between regions in the Edge Area, are unknown.

Road traffic and ‘found dead’ surveys have previously been used to study several wildlife diseases, including TB in badgers in England and Wales. Following the discovery of TB in badgers in the 1970s and 1980s^14–17^, several surveys of road-killed badgers for TB were undertaken in different areas of England and Wales (reviewed in Krebs et al.^18^). More recently, surveys of ‘found dead’ badgers in Northern Ireland and Wales have been published^19–21^, and also a study of road-killed badgers in Cheshire, NW England, in 2014^22^, a county that until 2018 straddled the Edge Area and the HRA. In each study, infection in badgers was found largely, although by no means totally, in areas in which TB in cattle was, at the time, most prevalent. Government agencies have also undertaken occasional small road-kill surveys around particular cattle outbreaks and found infected badgers, but we are not aware of these data having been published.

Ongoing long-term studies of badger TB in Gloucestershire (SW England)^23^ in the HRA, show long term persistence of TB in badger setts, and recent badger surveys around an isolated hotspot of TB in cattle in Cumbria (NW England), suggested that infection of imported origin in cattle was circulating in both badgers and cattle^24^. While these studies provide information about *M. bovis* in badgers and cattle in specific locations, there remains a lack of research into the relationship between *M. bovis* in cattle and badgers in other risk areas, particularly where the epidemic in cattle is spreading. Our study contributes to this knowledge gap.

**Figure 1:**
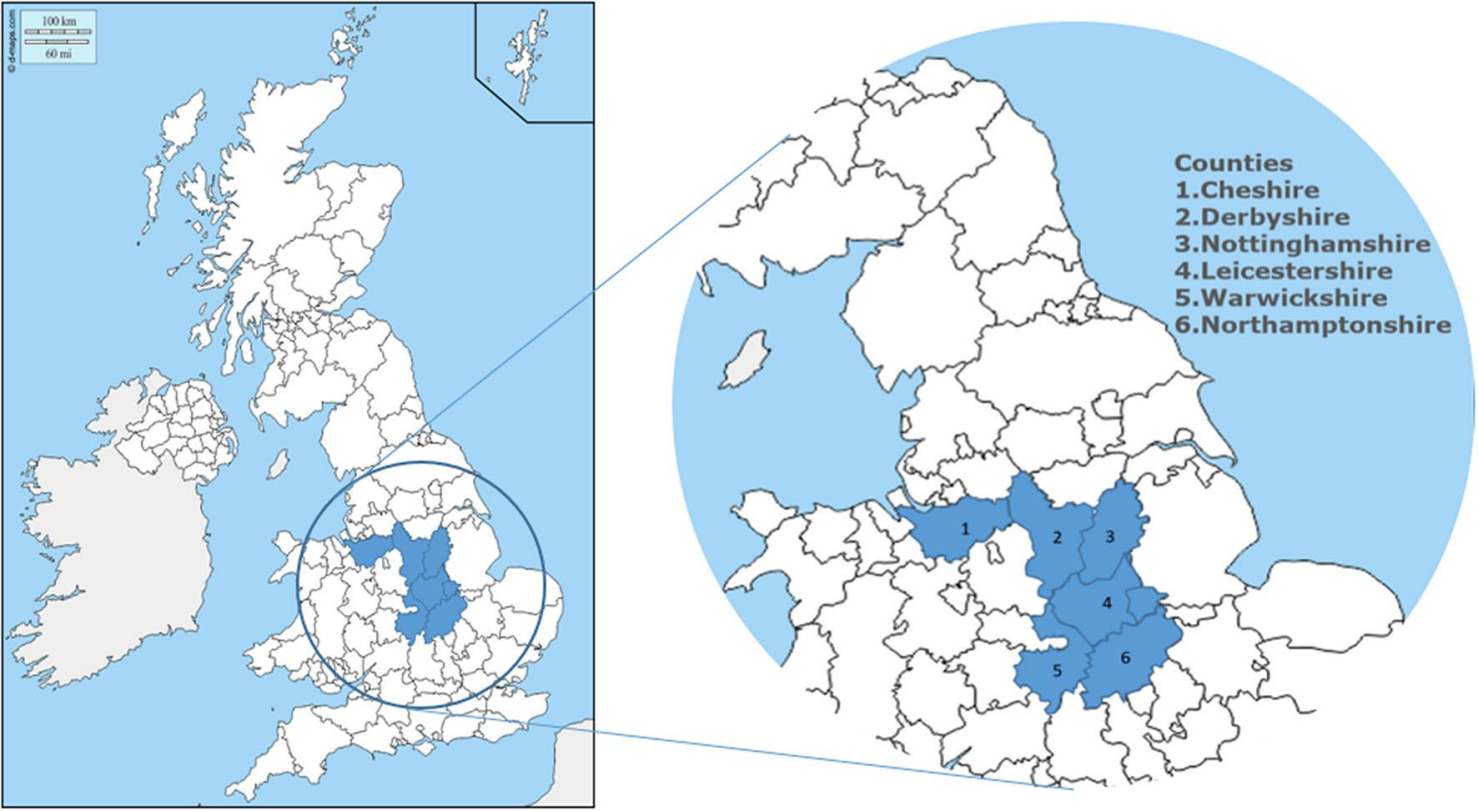
Map of the counties where RTA badgers were collected for testing.

Here we describe a study of road-killed badgers in northern Edge Area counties of England (Fig. 1; Cheshire, Derbyshire, Nottinghamshire, Leicestershire, Warwickshire and Northamptonshire), carried out in 2016–2017, and compare those findings with reported outbreaks of TB in cattle over the same area and time, collected by routine, government-regulated surveillance.

## Results

### Badger carcasses

**Figure 2:**
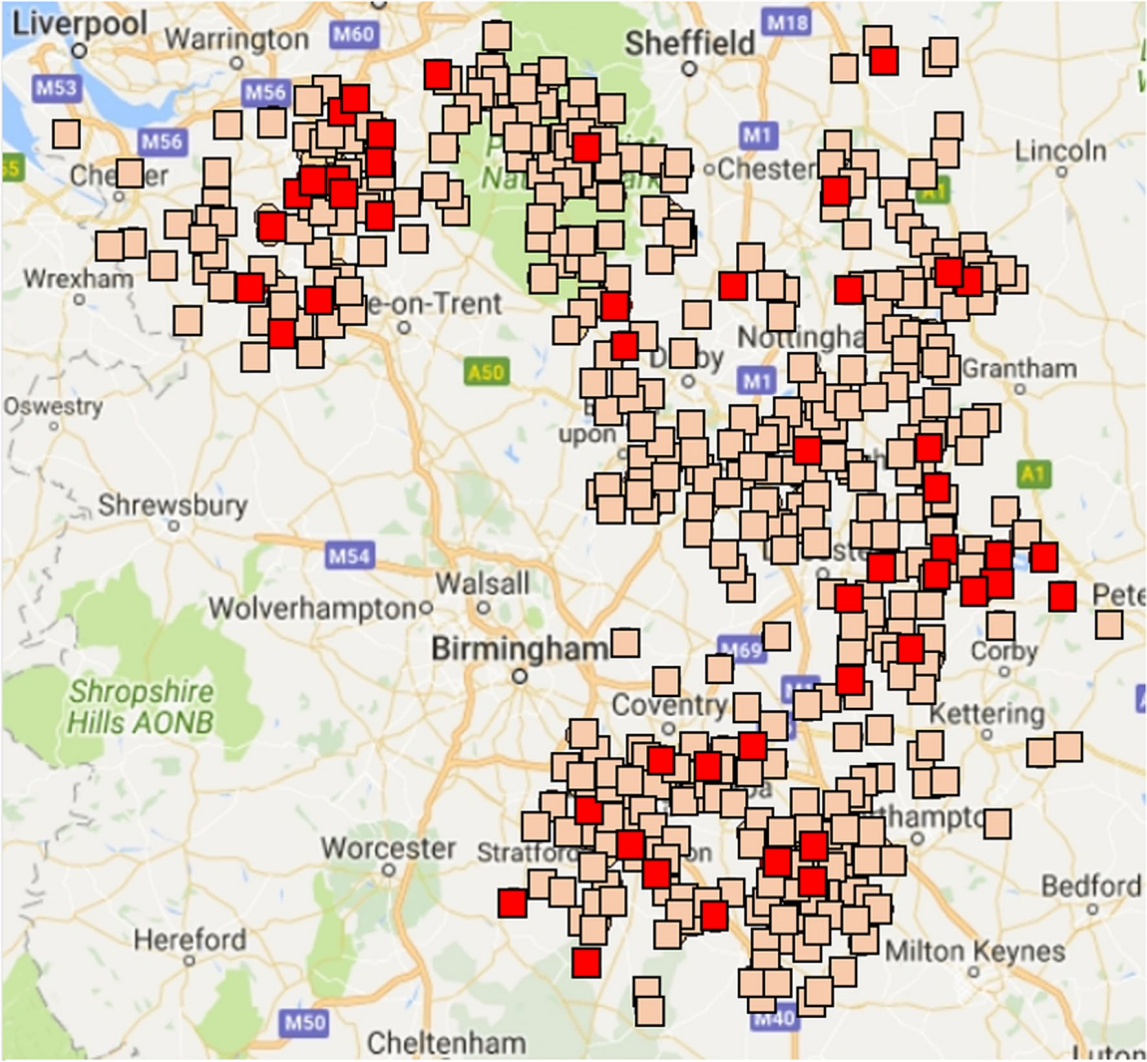
(**A**) the geographic distribution of badger carcasses sampled with those culture positive for confirmed Mycobacterium tuberculosis complex (MTC) in red and those negative in tan. (Original map generated in R-Googlemaps).

Overall, 610 badger carcasses suitable for necropsy and sampling, were collected from the study area (Fig. 2). Of these, 246 (40%) were collected by farmers and farming groups, 219 (36%) by badger or conservation groups and 145 (24%) by project staff, veterinarians, local authorities or other groups. These proportions differed by county (SI 1). The age and sex of the badger carcasses collected, when recorded (> 98% of carcasses), was overall 53% male and 46% female, and the age ratio 76% adult : 24% juvenile, with 13 (3%) of the adults classified as ‘old’ on the basis of pelage and tooth wear.

The availability of badger carcasses varied over time, with a peak in carcass collection in February 2017 (SI 1), and the number collected was capped at 100 badgers per county.

### Mycobacterium tuberculosis complex (MTC) in badgers

Overall, 51/610 (8.3%, 95% CI 6.4–11%) badgers were positive for MTC by culture and IS*6110* PCR. The prevalence of infection in males was approximately twice that in females (11% c.f. 5%, chi-square 6.019 p = 0.0142, odds ratio 2.16, relative risk 2.13). The prevalence did not vary significantly with the age of the badger, the time of year or the type of collector (‘farmer’, ‘wildlife’ group or ‘other’).

At post mortem examination, only one badger, which was MTC culture positive, had obvious, widespread lesions of TB, and another had atypical gross lesions from which MTC was isolated. The prevalence of MTC in the badgers varied between counties, such that Cheshire, Leicestershire and Warwickshire had higher prevalence than Derbyshire, Nottinghamshire and Northamptonshire (χ^2^ = 12.7, p = 0.026).

**Figure 3:**
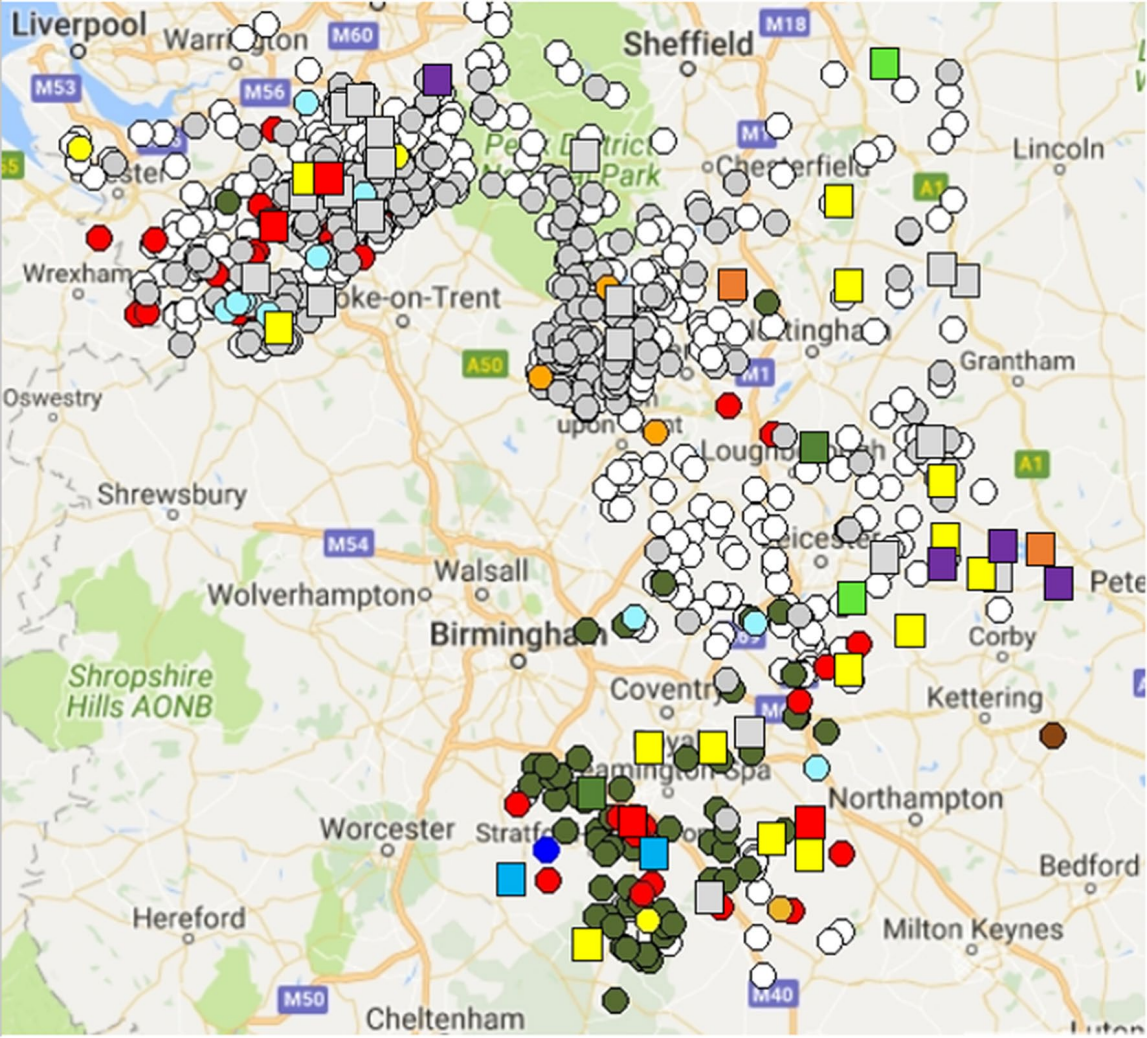
Overall distribution of spoligotypes of MTC isolates from northern EDGE Area cattle (circles) and badgers (squares) key: grey SB0129, red SB0263, blue SB0140, olive green SB0272, gold SB1016, orange SB1016, chocolate SB1414, green SB1512, yellow rare/untypable, purple M. microti. Cattle breakdowns with no known spoligotype are shown as white circles; these are usually breakdowns defined by skin tests but without MTC being cultured for typing. (Original map generated in R version 4.1.0 and package RgoogleMaps^40^).

Spoligotyping of badger MTC revealed a variety of spoligotypes, including those such as SB0129, SB0263, SB0272 and SB0140, frequently found associated with cattle infection in England^25^ but also a range of novel and rare spoligotypes (Figs. 2 and 3, and Supplementary Information). The most common spoligotype of *M. bovis* found in badgers (SB0129), was found particularly in the north of the study area, but also sporadically elsewhere. Other major spoligotypes of *M. bovis* isolated from cattle were found more sporadically in badgers.

### Mycobacterium tuberculosis complex bacteria in cattle

The results of farm-level surveillance testing of cattle for TB over the same period and area as the badger study are shown in Fig. 3. In total, there were 730,654 individual tests undertaken at 5,765 cattle holdings, and reported herd sizes ranged from 1 to 2,900 animals (median 62). There were 5227 positive animals, giving rise to 660 herd ‘breakdowns’ or outbreaks (11% of holdings) over the study period.

In the vast majority (91%) of herd breakdowns, the index case was detected by skin test during routine surveillance testing. When *M. bovis* was isolated from cattle, almost all (98.3%) of the spoligotypes from cattle were those previously identified in cattle in England. Each had a distinctive geographical range (Fig. 3) but some were also found sporadically in other regions. For example, the predominant spoligotype found in cattle during 2016–2017 was SB0129, particularly in the northern part of the study area, but the same spoligotype was also more sporadically found in other parts of the study area. Spoligotype SB0272 was the predominant spoligotype found in the southern part of the study areas, SB0263 was found sporadically throughout the study area, but mainly in western Cheshire.

### Comparison of MTC prevalence, spoligotypes and distributions in cattle and badgers

**Figure 4:**
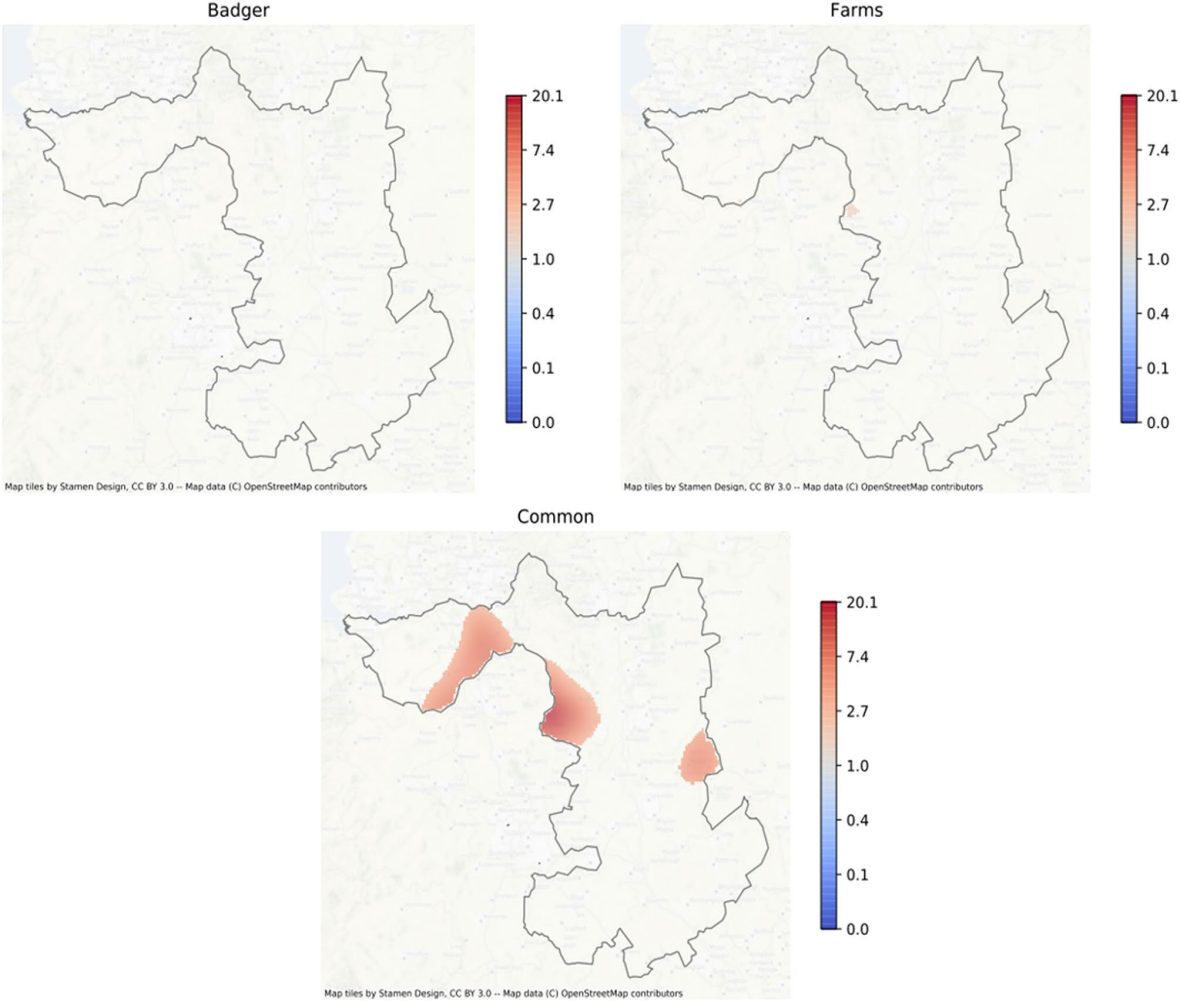
Mean posterior spatial odds ratio of MTC in the Edge region, highlighting regions of high posterior confidence that the spatial odds is above or below 1 (Pr(OR > 1) > 0.5 or Pr(OR < 1) > 0.5). For example, the common map indicates high prevalence foci in badgers and cattle in east, but not west Cheshire, in Derbyshire on the border with Staffordshire, but not in north-west Derbyshire, and in south Warwickshire. Areas of high prevalence for farms are seen in East Cheshire on the border with Staffordshire and in south Warwickshire. No areas of additional high prevalence are seen for badgers. Increase in red represents increased risk, and blue indicates decreased risk. Map tiles by Stamen Design, under CC BY 3.0. Data by OpenStreetMap, under ODbL.

**Figure 5:**
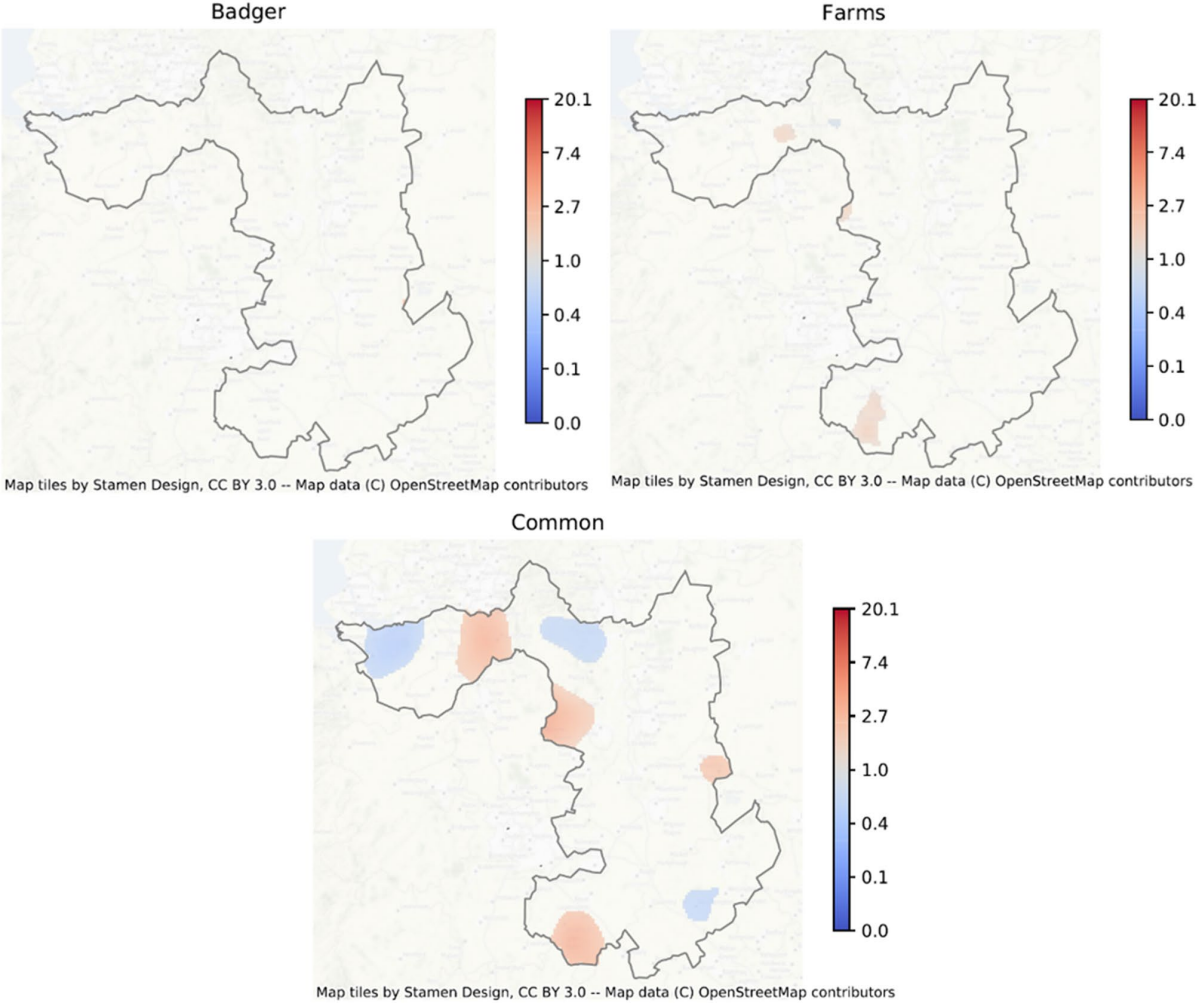
Mean posterior spatial odds ratio of SB0129 in the Edge region, highlighting regions of high posterior confidence that the spatial odds is above or below 1 (Pr(OR > 1) > 0.5 or Pr(OR < 1) > 0.5). The common map indicates high prevalence foci in Cheshire and Derbyshire and east Leicestershire. A small area of additional high prevalence for farms is seen in south west Derbyshire, bordering Staffordshire. No areas of additional high prevalence are seen for badgers. Increase in red represents increased risk. Map tiles by Stamen Design, under CC BY 3.0. Data by OpenStreetMap, under ODbL.

Spatially co-incident prevalence of MTC in badgers and on cattle farms was examined using a Bayesian bivariate logistic common-components geostatistical model^26^. Formal comparison of spoligotype distributions was only possible for SB0129 as the other spoligotypes found in badgers were too rare and sparsely distributed to be compared with those found in cattle. The spatial prevalence of both MTC and SB0129 was modelled in the badger and cattle (farm) populations using a combination of spatially-continuous risk surfaces (‘common’, ‘farm’, and ‘badger’), as well as a covariate adjusting for observation type (badger or farm). This enabled smoothed MTC and SB0129 risk surfaces to be fitted, describing how prevalence in farms and badgers varied in common over space, and also any additional departures from this common surface which might indicate spatial segregation of infection by observation type. The results, in terms of the posterior mean estimate of the spatial odds ratio for being MTC or SB0129 positive are shown in Fig. 4 (MTC regardless of spoligotype) and Fig. 5 (for SB0129). Only those regions where the posterior probability of the odds ratio is either greater or lower than 1 is at least 0.95 are plotted. For MTC (regardless of spoligotype), common high prevalence areas were seen in east Cheshire, in south west Derbyshire, on the border with Staffordshire, and in south Warwickshire, with areas of lower combined prevalence in east Cheshire, northern Derbyshire and central Northamptonshire. Similar areas of high prevalence in cattle were observed in east Cheshire, west Derbyshire and south Warwickshire, but no evidence of badger-specific foci was seen. Spatial comparison of SB0129 demonstrated obvious spatial foci of increased odds in east Cheshire and Derbyshire’ border with Staffordshire, but no pronounced evidence of additional cattle or badger-specific spatial foci was found for SB0129 (Fig. 5).

## Discussion

Stakeholders collected the badger carcasses and recorded their locations in this study, as described previously^22^. Although there was variation between counties, the type of stakeholder (farming group, wildlife group or other) was not overall associated with differences in the sex and age of badger carcasses collected or the prevalence of infection with MTC. Furthermore, the sex ratio, and proportions of animals by age were similar to those described in previous studies of live badgers^27,28^. Thus, while using road-killed badgers might involve biases in terms of space and time, there was no evidence that different stakeholder groups influenced the outcome of this study in terms of badger demographics or infection prevalence. As the density of badgers is not known in the Edge Area, and 100 carcasses were collected from each county (with counties of different areas), it is impossible to determine if heterogeneities in sampling density reflected heterogeneities in badger population density, stakeholder engagement or roads and road usage.

Overall, 51/610 (8.3%, 95% CI 6.4–11%) carcasses were culture positive and confirmed by PCR as MTC, a figure similar to that found for Wales in 2014–2016^21^. Previous studies found that the prevalence of infection in badgers was higher in adults than cubs, and in males than females, although in some cases these differences were not statistically significant^16,20,21,29^. This study found no significant difference in prevalence by age, although the numbers of, and prevalence in, younger badgers was small; but that the prevalence of infection in males was approximately twice that in females. It has been suggested this difference might reflect the larger home ranges of, and more territorial conflicts between, males compared to females, both of which could increase transmission^20,21^.

Most infected badgers had no macroscopic lesions suggestive of tuberculosis on post-mortem examination. The proportion of infected badgers with no obvious lesions (96%) was similar to that found previously in Cheshire^22^, but higher than that found in most studies of badgers in areas where infection is endemic in both cattle and badgers. The majority of positive cultures were from the ‘head and neck’, ‘thorax’ and ‘carcass’ pools of lymph nodes (27%, 27% and 21%, respectively). This is also similar to the findings of^22^, but contrasts with many previous studies, which found that isolation of *M. bovis* was most frequently from lung and thoracic lymph nodes^30,31^. These differences in infection sites and lesions may reflect differences sources of infection, in transmission routes and dose, or in the pathogenicity of the different genotypes of MTC found in the badgers across this study. It might also reflect differences in how carcasses were collected between these and other studies. Although it is often assumed that transmission of *M. bovis* among badgers is by the respiratory route, and requires lesions, transmission via bites is also thought to be important. It is conceivable that transmission via saliva and respiratory secretions through bites, grooming and other close contact, or, indeed, contaminated soil and bedding, associated with pharyngeal lymphoid infection is more likely, without the need for lesions. Indeed, such secretions might also enable shedding in faeces, with or without enteric infection.

There was variation in the frequency of infected badger carcasses between counties, with Cheshire, Leicestershire and Warwickshire having headline infection rates (10–15%) higher than Derbyshire, Nottinghamshire and Northamptonshire (4–5%). In Cheshire, 15/104 (13.5%, 95% CI 8–21%) badgers were confirmed MTC-infected, a prevalence not significantly different from that found in 2014^22^. There are no recent studies of MTC prevalence in badgers across the rest of the study area with which the results can be compared. Spoligotyping was performed rether than whole genome sequencing to enable comparison with historical cattle data. The most common spoligotype in MTC isolated from badgers was SB0129, particularly in the northern part of the study area where it was also the most frequent spoligotype found in cattle. Elsewhere, a wide range of spoligotypes were found in badgers, whereas in cattle the predominant spoligotypes were those expected if those infections resulted from expansion of regional cattle spoligotypes from neighbouring High Risk Area counties. The majority of cattle outbreaks in Leicestershire during the study were not confirmed by culture and so the spoligotype(s) responsible could not be identified. Subsequent surveillance reports (APHA, 2019), suggest that cattle outbreaks when spoligotyped remain largely of SB0129 with some suggestion of SB0272 expanding across the border from Warwickshire.

More formal analysis of the prevalence of MTC and spoligotypes in both cattle and badgers across the study area found no pronounced evidence of either MTC or SB0129 -specific spatial foci beyond those in eastern Cheshire and the Derbyshire border with Staffordshire, where SB0129 was and remains the predominant spoligotype found in cattle^32^. The approach used is robust to differing sampling densities of either farms or badgers across space, necessary with the inevitable variation in population density and sampling density. However, the diversity of spoligotypes found in badgers combined with the relatively low numbers of MTC-positive badgers in much of the study area, made further quantitative analysis difficult.

Thus overall, it appears that different transmission patterns may apply to different areas of the expanding edge of the TB epidemic in cattle in England. In the northwest of the study area, in Cheshire, there was evidence of persistence of infection in both badgers and cattle with the same spoligotype (SB0129), which together with an earlier study^22^ might suggest that infection in the two species is part of the same epidemic. Further east however, in Derbyshire and Nottinghamshire, there appeared to be an expanding epidemic of SB0129 in cattle, with little evidence of widespread infection in badgers, although few badgers were collected in the southwest corner of Derbyshire where TB has previously been described in badgers^33^. Where MTC-infected badgers were found, they were usually also infected with spoligotype SB0129. Further south in the study area, the prevalence of infection with MTC varied in both badgers and cattle, but whereas in cattle spoligotyping suggested an expanding epidemic from neighbouring High Risk Area counties, along with occasional long distance movements, a diversity of spoligotypes, including *M. microti* and occasional spoligotypes usually found in distant regions, was found in badgers. With the exception of in Cheshire, therefore, this study found little evidence to directly link the expanding epidemic in cattle in England to widespread badger infection. However, longer term and longitudinal studies will be required to understand better whether the expanding epidemics of regional spoligotypes in cattle are followed or led by similar dynamical changes in the prevalence of infection in badgers.

## Materials and methods

### Badger and sample collection

All badgers used in this study were found dead. The majority of badgers collected in this study were collected from road-sides and had lesions compatible with vehicle collisions. A small number were found dead in fields or farm buildings, some of which also had lesions compatible with vehicle collisions. The approach used was that developed for a previous study in Cheshire in 2014^22^: stakeholder groups collected carcasses, recorded where they were found, most often using smart phones, and the carcasses were then brought to the veterinary pathology facilities at either the University of Nottingham or University of Liverpool for necropsy and sampling. Stakeholders included farmers, veterinary practices, wildlife groups, local authorities and businesses as well as university staff. All experimental protocols were approved by the Defra and University of Nottingham.

Badgers were collected from six English counties in the northern edge of cattle TB epidemic (Cheshire, Derbyshire, Nottinghamshire, Leicestershire, Warwickshire and Northamptonshire) over a 16 month period from 2016 to 2017.

Where possible badger carcasses were stored in dry, cool areas or refrigerated. With two exceptions, frozen badgers were not included in the study. Carcass collection and necropsy occurred within 72 h of collection, and obviously autolytic or heavily damaged carcasses were not processed due to the likelihood of contamination. Each badger’s sex, weight and age (adult or juvenile, based on teeth, size and pelage) were recorded.

Lymph nodes and visible ‘TB-like’ lesions were sampled for culture. In addition to lung samples, four pools of lymph nodes were sampled: a ‘thoracic pool’ of bronchial and mediastinal lymph nodes, an ‘abdominal pool’ comprising hepatic and mesenteric lymph nodes, a ‘head and neck’ pool of parotid, mandibular, retropharyngeal and cervical lymph nodes, and a ‘carcass pool’ of prescapular, axillary, and superficial inguinal lymph nodes. If possible lesions were observed, each sample of lesion material was processed separately. Pooled tissues were stored at 4 °C for up to 48 h before processing. Individual tissue samples were stored at − 80 °C in case needed for further study.

### Tissue processing

Tissue samples for microbiological culture were processed in a Containment Level 3 (CL3) facility at the University of Nottingham, Sutton Bonington Campus. Samples were processed according to^22^. Briefly tissue pools or lesions were gently ground with sterile sand and 2 ml phosphate buffer saline (PBS; Dulbecco A, Oxoid). Samples were mixed with an equal amount of 5% oxalic acid (Fisher Scientific) and incubated at room temperature for 10 min to reduce non-mycobacterial contamination. The decontaminated pools (200 µl) were inoculated onto Stonebrink Selective agar supplemented with PACT (BD Diagnostics) and Middlebrook 7H11 agar slopes supplemented with PANTA (BD Diagnostics), and incubated at 37 °C for a minimum of 12 weeks. Cultures were examined approximately weekly for the appearance of colonies suggestive of mycobacteria.

### Characterisation of mycobacteria

Characteristic and possible colonies were picked from slopes after 12 weeks of incubation. Cells were heat killed (80 °C for 30 min) and DNA was extracted by crude heat lysis, in which heat-killed colonies were frozen and heated to 95 °C for 5 min in sterile distilled water (SDW), centrifuged (13,000 × *g;* 3 min) and the supernatant used as template DNA for subsequent PCR.

Isolates were screened for the MTB signature genetic element, IS*6110* by PCR^34^. For some colonies, the partial 16 s rRNA gene was amplified, sequenced (Source Bioscience, UK) and analysed to determine whether the colonies were part of the MTB complex or not^35^. DNA from confirmed members of the MTB complex were further interrogated by microarray-based spoligotyping (Alere Technology) according to^36^.

### Cattle data

Cattle testing data were obtained from APHA’s surveillance programme. At the time of the study, different cattle testing regimens were used in different counties. In HRA and Edge counties, cattle were routinely tested at herd level six-monthly or annually, with additional testing if infection was detected. Furthermore, some holdings had groups of cattle tested due to movements or because of epidemiological links to outbreaks detected elsewhere. Some TB-infected cattle were also detected during routine post-mortem inspection in the abattoir. As described in^22^, the original data sets from APHA included cattle holding location, the results of each test (including spoligotype if known), the official ‘breakdown identifier’ and start date, and the number of cattle on the holding and tested on each occasion. For the purposes of this study, all test results in the study period were combined for each site, and TB-positive herds were defined as those with at least one animal having tested positive using the ‘single intradermal comparative cervical tuberculin’ (SICCT) test, at least one animal with two consecutive inconclusive skin test results, positive interferon-gamma blood tests, or if lesions which were culture positive were found at the abattoir.

### Statistical analyses

The prevalence was estimated among the sampled badgers both overall and at a county level, as in previous studies^19,20^ on the assumption that the carcasses collected were representative of the overall population.

To detect evidence for co-occurrence of *MTC* positive badgers and SB0129 herd breakdowns, a bivariate Bayesian logistic common-components geostatistical model was used^37^. Observations consisted of a set of geographically located farms and badgers, with infection (breakdown) status recorded as a binary outcome variable (positive or negative), and observation type (farm or badger) as an explanatory variable. The outcome was modelled for each observation, *y*_*i*_, as a Bernoulli random variable with probability *p*_*i*_ such that

$$log\, it\left({p}_{i}\right)=\left\{\begin{array}{l}\alpha +\beta +{S}_{c}\left({x}_{i}\right)+{S}_{b}\left({x}_{i}\right)\quad if \, i \, is \, a \, badger\\ \alpha +{S}_{c}\left({x}_{i}\right)+{S}_{f}\left({x}_{i}\right)\quad if\, i \, is \, a \, farm\end{array}\right.$$

where *x*_*i*_ is the geographical location of observation *i*, and $\alpha$ and $\beta$ represent respectively the intercept and the log odds ratio for disease in badgers versus farms. The model makes use of three spatial Gaussian Processes, ${S}_{c}\left({x}_{i}\right)$, ${S}_{b}\left({x}_{i}\right)$, and ${S}_{f}\left({x}_{i}\right)$, characterised by a covariance function ${\Sigma }_{ijz}$ between the locations of observations *i* and j for “common”, “badger”, and “farm” spatial correlation components respectively. In each case, a Matérn correlation structure is assumed with sill and scale parameters ${\sigma }_{z}$ and ${\phi }_{z}$.^48^ Here, ${S}_{c}\left({x}_{i}\right)$ represents a disease risk surface common to both farms and badgers that allows disease prevalence to vary with space. Then, ${S}_{b}\left({x}_{i}\right)$ and ${S}_{f}\left({x}_{i}\right)$ represent respectively the *additional* spatial risk for badgers and farms respectively.

$${\Sigma }_{ijz}={\sigma }_{z}\left(1+\frac{\surd 3\Vert {x}_{i}-{x}_{j}\Vert }{{\phi }_{z}}\right)exp\left(\frac{-\surd 3\Vert {x}_{i}-{x}_{j}\Vert }{{\phi }_{z}}\right)$$

In a Bayesian context, prior distributions were assigned to the parameters as shown in Table 1.

**Table 1:** Prior distributions used for the parameters of the Bayesian logistic common-components geostatistical model.

Parameter	Prior distribution
$\alpha$	Normal(0, 100)
$\beta$	Normal(0, 100)
${\sigma }_{c}$, ${\sigma }_{b}$,${\sigma }_{f}$	Gamma(2, 2)
${\phi }_{c}$	Gamma(45, 2.5)
${\phi }_{b}$, ${\phi }_{f}$	Gamma(10, 2)

In contrast with previous work in Cheshire^22^, the volume of data analysed here (5522 farm and 565 badger locations) precluded a naïve implementation of the three Gaussian processes. Instead, a low-rank approximation was used in which a number of “knot” points were chosen to represent the Gaussian process in 2-dimensional space, and the resulting representation projected onto the observation points. Here, 600 and 200 points were chosen for farms and badgers respectively by 2-dimensional k-means clustering^38^.

The model was implemented in Python 3.6. using the PyMC3 3.7 package^39^, with the No-U-Turn Sampler (NUTS) used to draw samples from the joint posterior distribution. The sampler was run for 20,000 samples, discarding the initial 1500 as tuning and burn-in. The convergence of 4 MCMC chains was inspected visually and found to be satisfactory. Traceplots for these runs are shown in Supplementary Fig. 1 together with Gelman-Rubin statistics demonstrating convergence ($\widehat{R}$ in all cases).

To detect co-regionalisation of infection in both badgers and farms, the Bayesian predictive distribution of the Gaussian Process was evaluated on a fine grid over the study area^38^. To summarise each predictive distribution, both the mean posterior value of the Gaussian process, and the probability that the posterior distribution at each grid point was greater or less than zero, were calculated. Since the Gaussian process represented the spatial log odds ratio, this is equivalent to calculating the posterior probability of the spatial odds ratio being greater or less than 1, i.e. Pr(OR > 1) and Pr(OR < 1) respectively, noting that Pr(OR > 1) = 1 – Pr(OR < 1). To present the results, we plot maps of the mean posterior spatial odds ratio for pixels where Pr(OR > 1) > 0.95 or Pr(OR < 1) > 0.95. Thus our maps highlight regions where there was good evidence that the odds ratio is significantly different to 1 (i.e. no spatial effect).

## Supplementary Information

Supplementary Information 1.

Supplementary Information 2.
