# Supplementary material for: Tuberculosis in badgers where the bovine tuberculosis epidemic is expanding in cattle in England
Source: Sci Rep. 2021 Oct 25;11:20995. doi: 10.1038/s41598-021-00473-6 (PMC8545939; doi:10.1038/s41598-021-00473-6)
Supplement: Supplementary file 2 — Supplementary Information 2. [file 41598_2021_473_MOESM2_ESM.pdf]

Supplementary Figure 1: Convergence diagnostics for MCMC

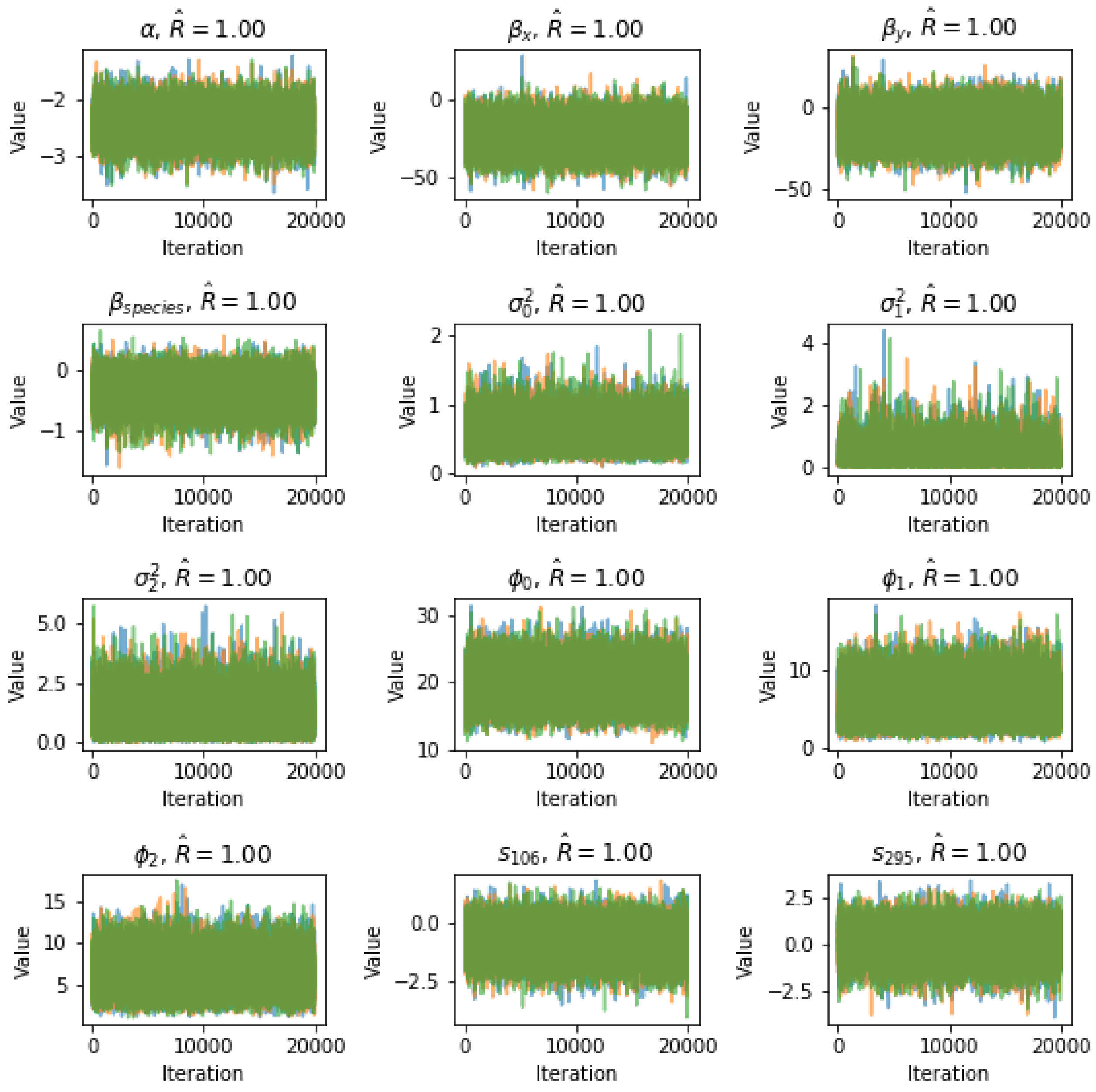

Supplementary Figure 1: Superimposed traceplots for all model parameters and two randomly-chosen inducing points (s) showing satisfactory MCMC convergence. In addition, Gelman-Rubin (Gelman and Rubin, 1992) statistics (R) are provided and are all close to the expected value of 1.
